# Chronic Venous Disorders: The Dangerous, the Good, and the Diverse

**DOI:** 10.3390/ijms19092544

**Published:** 2018-08-28

**Authors:** Daniela Ligi, Lidia Croce, Ferdinando Mannello

**Affiliations:** Department of Biomolecular Sciences, Section of Clinical Biochemistry and Molecular Genetics, University “Carlo Bo”, 61029 Urbino, Italy; daniela.ligi@uniurb.it (D.L.); lidia.croce@uniurb.it (L.C.)

**Keywords:** chronic venous disorders, inflammation, venous leg ulcer, wound healing, matrix metalloproteinases, biomarkers, cytokines, glycosaminoglycan, glycocalyx, chronic venous insufficiency

## Abstract

Chronic venous disorders are common vascular pathology of great medical and socioeconomic impact, characterized by a wide spectrum of clinical manifestations occurring with symptoms and/or signs that vary in type and severity. The predominant pathophysiological mechanisms of chronic venous disease start from the development of venous hypertension from shear stress and reflux, leading to endothelial dysfunction and venous wall dilatation. The altered hemodynamic transduces physical signals into harmful bio-molecular pathways, creating a vicious cycle among shear stress, proteolytic remodeling, and inflammatory processes. This intricate network is further exacerbated by the degradation of protective endothelial glycocalyx. In this special issue, at least three main aspects of these interactions are highlighted: the dangerous, the good, and the diverse, which may help to focus attention on the biomolecular mechanisms and the possible targeted therapy of chronic venous disorders (CVeD).

## 1. Introduction

Chronic venous disorders (CVeD) are common lower extremity vascular pathologies of great medical and socioeconomic impact (1–2.5% of health care budgets in developed countries), affecting a large part of the population worldwide (prevalence as high as 73% in women and 56% in men) and significantly decreasing the quality of life of affected patients [1]. 

CVeD represents the sequelae of a general venous insufficiency that spans a wide spectrum of clinical manifestations from varicose veins to edema to skin changes to the development of venous ulceration. In fact, venous diseases include clinically deteriorating conditions with morphological and functional alterations of the venous system, which occur with symptoms and/or signs that vary in type and severity as highlighted by the clinical, aetiological, anatomical, and pathological (CEAP) international classification [2].

The predominant pathophysiological mechanism of chronic venous insufficiency (CVI) is the development of venous hypertension from shear stress and reflux of incompetent valves [3]. Venous hypertension can lead to venous dilatation, worsening valve insufficiency, and generation of a perpetuating cycle of increasing pressure and further dilation. The changes in hemodynamics are transmitted to the microcirculation, to endothelial cells and vessel microenvironment leading to venous microangiopathy, with dilation and tortuosity of capillary beds [3]. The mechano-sensors of endothelial cells, triggered by the altered hemodynamic, transduce physical signals into harmful bio-molecular pathways resulting in endothelial damages. In particular, these complex biological processes activate inflammatory and proteolytic cascades in the vascular microenvironment, including leukocyte adhesion, degranulation, and release of cytoplasmic granules from neutrophils, macrophages, mastocytes, endothelial cells, and platelets [4]. 

All these steps lead to the formation of a deleterious network and a vicious cycle impairing micro- and macro-circulatory flow, creating an environment that causes remodeling of the vein walls and valves, venous hypertension, formation of varicosities, edema, and leg ulceration [3,5].

It is noteworthy that all the medicinal products able to combat the initiation and development of endothelial inflammation and limit the excessive proteolysis are of fundamental importance for treating all the CVeD stages, starting from the less severe (but significant starting point, such as telangiectasia and varicose veins) up-to the highly invalidating active ulcer and dangerous pre- and post-thrombotic conditions [5,6]. 

The sum of all research endeavors on CVeD will lead to an increased, detailed, and more complex understanding of the multidimensional interactions that hemodynamic alterations keep with biomolecular signaling in CVeD. In this special issue, through original research articles and reviews improving our knowledge on the pathophysiology of CVeD, at least three main aspects of these interactions are highlighted: the dangerous, the good, and the diverse, which may help to focus attention on the biomolecular mechanisms and the possible targeted therapy of CVeD.

## 2. The Dangerous: The Chronic Hemodynamic Alteration and Molecular Transduction of Deleterious Biofactors in Venous Microenvironment

**Figure 1:**
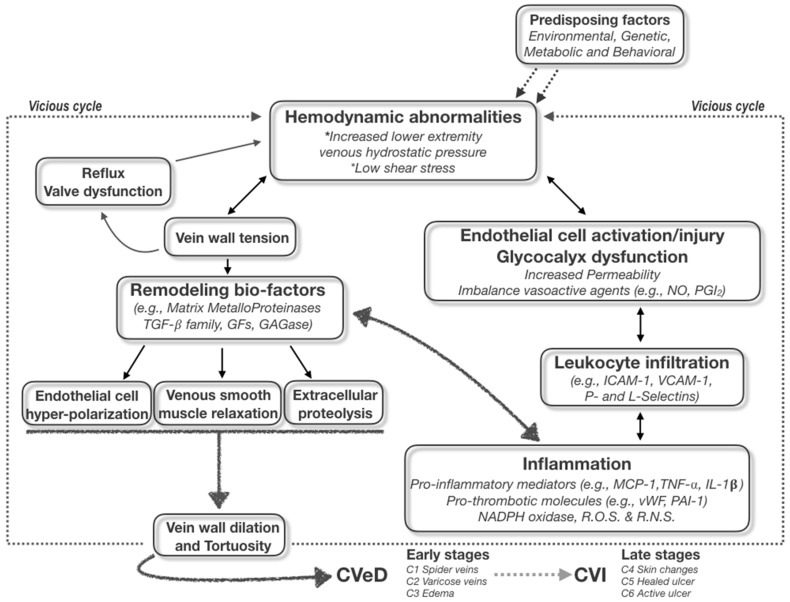
The Dangerous network of the complex mechanisms contributing to both initiation and progression of the different stages of CVeD.

It is well known that some genetic, environmental, and behavioral risk factors (e.g., family history, seasonality, and standing up position/work activities) may cause an increase in hydrostatic pressure, leading to valve disfunction and then venous reflux. The symptoms of vein disorders are biochemically and molecularly mediated by the inflammatory responses of the venous wall; in particular, the increased permeability and the passage of different types of leukocytes from the circulatory system to the extra-cellular matrix favor the onset of swelling, heaviness, and pain [2,3]. This starting point begins from the post-capillary venules of the microcirculation (where the inflammatory burden is concentrated under the conditions of high hydrostatic reflux pressure) and then is transferred to larger vessels and transduced to the damaging signaling of both endothelial wall and venous valves through the infiltration of leukocytes in peri-venous microenvironment [4]. The primum movens of the vicious cycle is then originated, boosting the CVeD chronic status and leading to the varicose vein development and progression to ulcer and thrombotic risks (Figure 1).

The first issue of the disease becoming chronic is therefore central to the practical clinical approach to CVeD; it should be emphasized that all CVeD clinical pictures are connected to each other because they have a common pathophysiological biomolecular mechanism(s), in which the crucial events of hemodynamic alterations pave the way for a self-sustained vicious cycle of subsequent inflammatory and proteolytic cascades [1,3].

These harmful processes create in varicose veins some typical atrophic regions in vein wall, in which a degradation of extracellular matrix and an increased infiltration of inflammatory cells significantly occur [5]. In fact, collagen fibers appear disorganized and the elastic fibers appear thick and proteolytically fragmented. Interestingly, in the early stages of CVeD it has been demonstrated a significant activation and variability of expression of several MMPs, both at biochemical and molecular levels [7]. These observations support the theory that the dilation of the vein walls (also due to the proteolytic digestion of elastin and other matrix components of the vessel wall) may represent a primary pathological event [8], mainly due to distortion and dysfunction of the venous valves, capable to leading over time to venous reflux, valvular incompetence leads to ambulatory venous hypertension, thus favoring the appearance of varicose veins observable with the clinical examination [2]. 

Regardless of the “primary” pathological event, both venous wall dilation and venous valve dysfunction appear to contribute to the pathogenesis of varicose veins and to the changed flow hemodynamics, primum movens for establishing inflammatory/remodeling-based vicious cycle within vein microenvironment (Figure 1). In this respect, it is noteworthy to highlight the presence inside the vein wall and microenvironment of a series of metabolic activities involving ions (e.g., Ca^2+^, Zn^2+^, and Fe^2+^), signaling molecules (e.g., growth factors and reactive oxygen/nitrogen species) and enzymes (e.g., NADPH oxidase, plasminogen activator, serine proteases, heparanase, glycosaminoglycanases, and MMP) [3,5,7]. 

For what concerns the function of growth factors, it has been clearly demonstrated that Transforming Growth Factors, in particular TGF-β family, play a crucial role in the vascular wall physio-pathology, in both early (C2) and late (C6) stages of CVeD [9]. In fact, different TGF-β isoforms and their signaling receptors may actively participate in the MMP/TIMP imbalance throughout the CVeD progression [7], inducing the cleavage and release of soluble Endoglin, a well-known protein antagonizing TGF-β signaling a crucial process in vascular pathologies [10].

The proteolytic enzymes MMPs are widely known for their ability to degrade almost all the extracellular matrix proteins, including the selective degradation of the vein wall elastin and the glycosaminoglycan protective layer of endothelium, allowing the harmful interaction of leukocytes with endothelial cell membrane, influencing thus proliferation, migration, and differentiation processes (e.g., apoptosis, immune response, tissue remodeling, and angiogenesis) [11]. 

**Figure 2:**
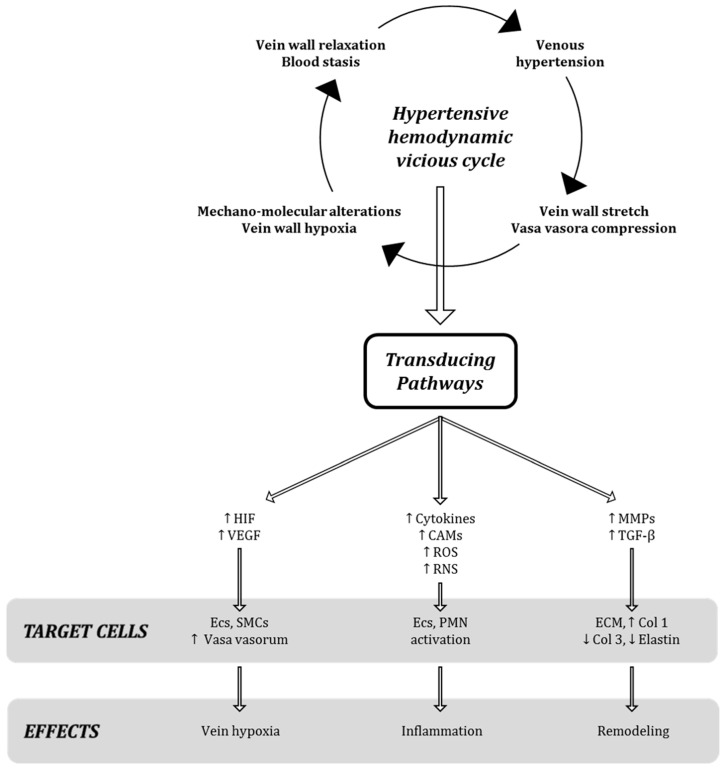
Schematic representation of biomolecular transduction pathways related to the hypertensive hemodynamic alteration in early stages of CVeD and late steps of CVI (modified and adapted from ref. [4]).

It is well known that the increase in venous pressure is also able to enhance the tension on the endothelial wall, which in turn stimulates the production and secretion of growth factors, cytokines and proteinases, as specific molecular events after mechano-transduction stimuli in venous milieu (Figure 2) [4].

The enhanced production, secretion, and activation of several enzymes (including heparanase, matrix metalloproteinases, and glycosaminoglycanases) [11] induced by venous pressure leads to the destruction of the protective layer of glycocalyx from the surface of endothelial cells, paving the way for the vessel bio-damage [3,5] and the significant morphologic changes of vessel wall.

## 3. The Good (and Fragile): Glycosaminoglycans Positively Affecting and Protecting Blood Vessels

The properties and functions of glycosaminoglycan (GAG) layer, forming a protective barrier in endothelial cells, are critical for the maintenance of water, ions, and protein balance between the intra- and extra-vascular compartments [12]. The chemical modification and/or loss of glycocalyx endothelial barrier have been implicated in the genesis and/or progression of a variety of pathological conditions, including venous diseases. The altered barrier function in these conditions is even more related to the active extra-cellular release of soluble bio-compounds from resident cells and/or recruited blood cells (e.g., neutrophils, mast cells, macrophages, and lymphocytes) [13]. In fact, the loss of endothelial glycocalyx by increased shear stress or hydrostatic pressure as well as its peculiar degradation by some proteolytic enzymes (e.g., matrix metalloproteinase, elastase, heparanase, etc.), allows the interaction of the activated blood cells with receptors expressed on the surface of endothelial cells, diminishing the barrier function and facilitating extravasation of reactive “leukocytes” [3,14]. 

The highly negatively charged, unbranched polysaccharide family of GAGs (like heparin, heparan sulfate, and dermatan sulfate) may mediate chemokine mobilization, modulating the recruitment of leukocytes and neutrophils, a vital process in inflammation [12]. Interestingly, it has been widely demonstrated that both macrophage and neutrophil transmigration in vitro can be reduced by adding soluble GAGs and that this process is specific with respect to the nature of the glycan composition [15]. 

In vitro and in vivo studies have highlighted that the GAGs of endothelial glycocalyx have a profound influence on the vascular wall physiology through: (1) the transmission of shear stress, as mechano-transducer of some hemodynamic stimuli; (2) the maintenance of a selective permeability barrier and a low hydraulic conductivity, like a protective layer for the endothelial cells, both in arteries and veins; and (3) the modulation of blood leukocytes, lymphocytes, and platelets adhesion, limiting extravasation of reactive cells capable of inducing inflammation and proteolysis in blood vessel microenvironment. So, endothelial glycocalyx (and its GAG constituents) represents the good but fragile barrier of both vein and artery wall [16].

The major constituents of the endothelial glycocalyx may be degraded from the endothelial surface under various acute and chronic clinical conditions; a plethora of proteolytic enzymes may have a wide array of potentially destructive capability against vessel GAGs. In fact, other than the well-known matrix metalloproteases, also heparinases, hyaluronidase, and serine proteases (like thrombin, elastases, and plasminogen), as well as cathepsins, may lead to loss or destruction of GAG from the endothelial surface layer [14]. All these proteinases may be released or actively secreted during altered hemodynamic conditions in both veins and arteries, representing a possible target for future therapeutic approaches reviewed in [6,17]. 

In this respect, pharmacological agents (such as inhibitors of inflammation and inhibitors of excessive proteolytic activity, like glycosaminoglycans and purified flavonoids) [6] may display potential to attenuate shedding of the glycocalyx in various experimental models [17]. Several clinical evidences and in vitro studies on CVeD (and its thrombotic complications) suggest a benefit from the therapeutic use of the GAG-based drug sulodexide, a preparation delivering highly purified mixture of precursors of the glycocalyx constituents, like heparin and dermatan sulfate [6,18]. In recent years, this medicinal product has demonstrated an anti-inflammatory, anti-proteolytic, and anti-thrombotic actions on the endothelial wall in patients suffering of CVeD at different stages of disease progression, suggesting it in the Guide Lines as promising approach for the management of vascular diseases [19,20].

## 4. The Diverse: Elucidating Mechanistic Strategies for Targeted Therapy

**Figure 3:**
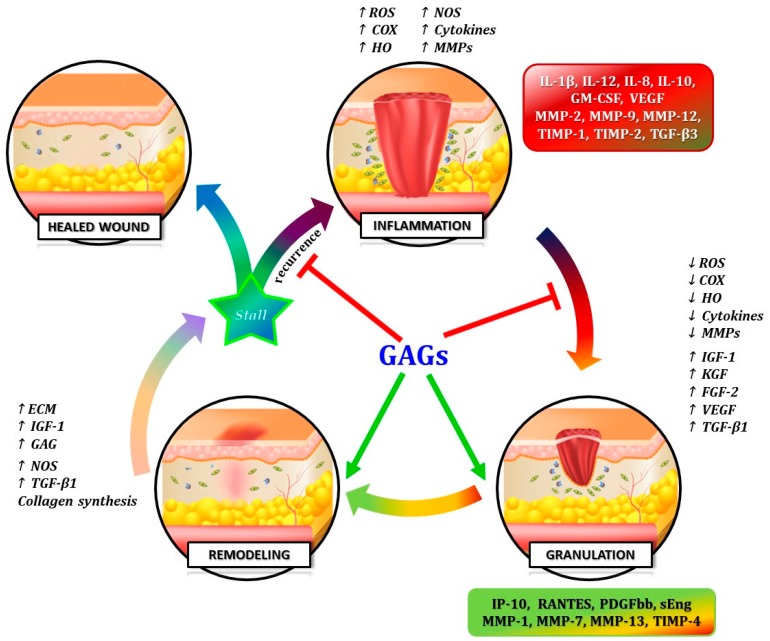
Schematic representation of the ability of GAGs, like Janus Bifrons, to down-regulate the harmful inflammatory/proteolytic pathways (red T-bar) and to up-regulate the favorable anti-inflammatory/proteolytic cascade (green arrows) in venous microenvironment during the different stages of CVI. “Stall” represents the critical condition, like a “biological switch”, in which the chronic venous disorders may enter either in the inflammatory vicious cycle (recurrence of delayed healing of leg ulcers) or progress to the enhanced healing processes of the wounds.

The glycosaminoglycans physiologically present as a protective layer and a significant barrier for all endothelial cells [12], as well the commercially available GAG-based drugs, represent biomolecular compounds showing a double but complementary function, acting like “Janus Bifrons” [18]. Through the modulation of a wide variety of bio-compounds with opposite biologic activities, GAGs can limit harmful inflammatory and proteolytic cascade and enhance anti-inflammatory pathways and tissue remodeling [15,21] (Figure 3). 

Several studies provided evidence that the integrity of GAG compounds of endothelial glycocalyx plays crucial roles and protective functions in several vascular diseases. The loss and/or degradation of glycocalyx prone the vessel wall to the development of inflammatory and proteolytic damages “opening the door” for: (a) the mechano-transduction of acute and chronic stimuli; (b) the increased permeability barrier; (c) enhancing harmful adhesion of blood leukocytes, lymphocytes, and platelets; and (d) favoring extravasation of the reactive blood cells capable of induce inflammation and proteolysis in blood vessel microenvironment [1,3,14].

On the other hand, studies on the GAG-based drugs have demonstrated its “Janus Bifrons” vascular protective properties [18], through down-regulation of pro-inflammatory cytokines and up-regulation of anti-inflammatory interleukins [22,23], anti-thrombotic action and pro-fibrinolytic property, and overall the capability of replenishing GAGs in vessel wall [20] (Figure 3). 

Several veno-active drugs (including mainly GAG-based compounds and also purified flavonoids) may counteract the hemodynamic, inflammatory, and proteolytic pathologic mechanisms of venous diseases, including all stages of CVeD [6,17] but also venous thromboembolism complications [24]. 

The potential of these veno-active and veno-protective drugs is mainly focused to limit the initiation/progression of early stages of CVeD, accelerating also the wound closure in the late stages of CVI, suggesting then a potential utility of GAG-based drugs in endothelial dysfunctions as a promising therapeutic approach for patients suffering of both deep-vein thrombosis and pulmonary embolism, serious and complicated pathologies with significant clinical and epidemiological impact in the elderly reviewed in [24].

## 5. Conclusions

An updated consensus highlighted all the clinically complex aspects included in the terminology Chronic Venous Disorders (CVeD), including early clinical pictures which cannot be called simply “disease” (e.g., teleangiectasias) compared to the late stages CVI mainly due to insufficient venous drainage, edema, skin damage, and leg ulcers [25]. This clinical differentiation appears important since venous hypertension (which is termed the main driving force for the described biochemical changes) does not play a role in all CVeD stages. In fact, before venous hypertension occurs, there are some biological mechanisms involved (e.g., early features like leukocyte adhesion to endothelial cells, capillary fenestration, early stages of inflammatory signaling due to mechanosensory transduction, etc.) which are at the border between normal physiology and pathology, probably the “switch” driving and leading to the late mechanisms occurring in the venous microenvironment (e.g., hypertension, shear stress, venous dilatation, and endothelial dysfunction). The complex biomolecular events (e.g., inflammatory, proteolytic, and thrombotic pathways) occurring in all stages of the CVeD, as well the diverse clinical pictures associated with the different stages of CVeD, seem to have a common physio-pathological basis. According to the original hypothesis [8], the early inflammatory events (e.g., increased capillary filtration, endothelial cell fenestration, leukocyte transcapillary penetration) as well the late hemodynamic processes (relaxation, reflux, and venous hypertension) may provoke endothelial dysfunction (especially in subcutaneous areas) and worsen the inflammatory responses. 

It is noteworthy that for resolving CVeD we need to take into account different approaches: on one hand, the ability of pharmacological therapy to target the dangerous biochemical events, on the other hand the positive role of the physical “anti-stasis” effects of both compression and/or surgical reflux ablation.

The first endothelial alterations induce and guide the subsequent harmful inflammatory and proteolytic cascades, mainly evidenced by the classic hemodynamic CVeD symptomatology (like swollen, heavy, painful legs connected to increased vessel wall permeability, leukocyte infiltrations, and pathological remodeling of the venous wall).

Starting from the primum movens, the complicated network of the CVeD is further exacerbated by the vicious cycle, giving life, over time, to progressive pictures of varying severity, from telangiectasia to varicose veins (typical of CVeD), to edema and cutaneous trophic lesions up-to venous ulcers (characteristics of CVI), including also venous thromboembolism [3,8]. It is noteworthy that the late stages of CVeD, mainly identified as CVI, are frequently caused by a post-thrombotic syndrome, which represent a classical model for an interesting interplay between biochemical mechanisms in the blood and the vessel wall, and in which glycosaminoglycan-derived drug and compression may play crucial roles and effects.

An understanding of the hemodynamic-inflammatory-proteolytic biomolecular mechanisms and events may help to orientate the treatment in a more targeted way to the needs of the individual patient suffering of CVeD [6,17]. The use of GAG-based drugs represents a key point in the treatment of both CVeD and CVI for the inflammatory and proteolytic aspects of symptoms and thrombotic risk [19]. 

Finally, GAG-based drugs represent the good and the diverse to counteract the dangerous network among hemodynamic, inflammatory, and proteolytic processes occurring from the early stages of symptomatic varicose veins to severe steps of ulcers including the post-thrombotic patients, stigmatizing the fundamental role of the biomolecular mechanisms of endothelium in the management of vascular diseases.

## Abbreviations

CAM	Cell-adhesion molecule
CEAP	Clinical, Etiological, Anatomical, and Pathophysiological classification
*COL-1/3*	Collagen-1/3 genes
COX	Cyclo-oxygenase
CVeD	Chronic venous disorders
CVI	Chronic venous insufficiency
EC	Endothelial cell
ECM	Extra-cellular matrix
FGF-2	Fibroblast growth factor-2
GAG	Glycosaminoglycan
GAGase	Glycosaminoglycanase
GF	Growth factor
HIF	Hypoxia-inducible factor
HO	Hemeoxygenase
IGF-1	Insulin growth factor-1
IL	Interleukin
KGF	Keratinocyte growth factor
MCP-1	Monocyte chemoattractant protein-1
MMP	Matrix metalloproteinase
NO	Nitric oxide
NOS	Nitic oxide synthase
PAI-1	Plasminogen-activator inhibitor-1
PDGF-bb	Platelet derived growth factor-bb
PGI-2	Prostaglandin I-2
PMN	Polymorphonuclear cells
RNS	Reactive nitrous species
ROS	Reactive oxygen species
sENG	Soluble Endoglin
SMC	Smooth muscle cell
TGF-β	Transforming growth factor-beta
TIMP	Tissue inhibitor of metalloproteinase
TNF-α	Tumor necrosis factor-α
VEGF	Vascular endothelial growth factor
vWF	von Willebrand factor
